# Veterinary Hospital Dissemination of CTX-M-15 Extended-Spectrum Beta-Lactamase–Producing *Escherichia coli* ST410 in the United Kingdom

**DOI:** 10.1089/mdr.2016.0036

**Published:** 2016-10-01

**Authors:** Dorina Timofte, Iuliana Elena Maciuca, Nicola J. Williams, Andrew Wattret, Vanessa Schmidt

**Affiliations:** ^1^ School of Veterinary Science, University of Liverpool, Liverpool, United Kingdom.; ^2^ Department of Infection Biology, Institute of Infection and Global Health, University of Liverpool, Liverpool, United Kingdom.; ^3^ Faculty of Veterinary Medicine, University of Agronomical Sciences and Veterinary Medicine, Iasi, Romania.; ^4^ Department of Epidemiology and Population Health, Institute of Infection and Global Health, University of Liverpool, Liverpool, United Kingdom.

**Keywords:** *E. coli*, ESBL, surveillance, veterinary, infection control

## Abstract

We characterized extended-spectrum beta-lactamases (ESBLs) and plasmid-mediated quinolone resistance (PMQR) in 32 *Escherichia coli* extended spectrum cephalosporin (ESC)-resistant clinical isolates from UK companion animals from several clinics. In addition, to investigate the possible dissemination of ESBL clinical isolates within a veterinary hospital, two ESBL-producing *E. coli* isolates from a dog with septic peritonitis and a cluster of environmental ESC-resistant *E. coli* isolates obtained from the same clinic and during the same time period, as these two particular ESBL-positive clinical isolates, were also included in the study. Molecular characterization identified *bla*_CTX-M_ to be the most prevalent gene in ESC-resistant isolates, where 66% and 27% of clinical isolates carried *bla*_CTX-M-15_ and *bla*_CTX-M-14,_ respectively. The only PMQR gene detected was *aac(6')-Ib*-*cr*, being found in 34% of the ESC *E. coli* isolates and was associated with the carriage of *bla*_CTX-M-15_. The clinical and environmental isolates investigated for hospital dissemination had a common ESBL/AmpC phenotype, carried *bla*_CTX-M-15_, and co-harbored *bla*_OXA-1,_ *bla*_TEM-1,_ *bla*_CMY-2,_ and *aac(6')-Ib*-*cr.* Multilocus sequence typing identified them all as ST410, while pulse-field gel electrophoresis demonstrated 100% homology of clinical and environmental isolates, suggesting hospital environmental dissemination of CTX-M-15–producing *E. coli* ST410.

## Introduction

*E**scherichia coli* are opportunistic pathogens in humans and companion animals and can be associated with a variety of extraintestinal infections, which may require antimicrobial therapy.^1^ Increased use of antimicrobials in companion animals may select for antimicrobial-resistant bacteria, and concerns have been raised that these host species may act as potential reservoirs for human infections.^2^ Particularly concerning for both human and veterinary health is the increasing resistance to extended spectrum cephalosporins (ESCs) through the production of extended-spectrum beta-lactamases (ESBLs).^3–6^ There is increasing evidence that *E. coli-*producing ESBL and/or AmpC beta-lactamases are emerging in companion animals,^7,8^ setting new challenges for veterinary practitioners due to therapeutic and infection control implications.

Several studies have described the prevalence of ESBL resistance in bacteria from companion animals,^3,4,9–11^ but the role that these animals may play in the spread of such resistant bacteria or determinants, is not yet fully determined. Previous molecular studies have shown that, in human hospital settings, ESBL genetic determinants have the potential to spread either through clonal dissemination or plasmid transfer, posing a serious threat to patient care and safety.^6,12^ The development of large veterinary hospitals with intensive care facilities has created similar conditions for the emergence of animal hospital acquired infections and a few studies have shown the association of multidrug resistant (MDR) organisms, such as *E. coli, Acinetobacter baumannii, Enterobacter* spp., and *Enterococcus* spp., with animal nosocomial infections.^13–19^ However, with the exception of a few recent studies showing the hospital acquisition and/or dissemination of beta-lactamases or ESBL-producing *Klebsiella pneumoniae* and *A. baumannii*^20–23^ and compared with the wealth of data from human medicine, there is a paucity of studies investigating the potential of ESBL-producing *E. coli* to spread and cause nosocomial infections in veterinary clinics or hospital settings.

The aim of this study was dual; first, to characterize ESBL and plasmid-mediated quinolone resistance (PMQR) genes in *E. coli* from clinical specimens submitted for routine bacterial culture to the Veterinary Microbiology Diagnostics laboratory in the Liverpool School of Veterinary Science. Second, to analyze and compare a cluster of clinical and environmental ESBL-producing *E. coli* obtained from a UK Veterinary Hospital to identify the potential of clinical isolates to spread within such environments.

## Materials and Methods

### Bacterial isolates

All *E. coli* isolates were obtained from companion animal clinical specimens submitted from a veterinary hospital and a number of small veterinary clinics and collected for this study between January 2010 and November 2011. Clinical specimens were plated out aerobically on 5% sheep blood agar (Oxoid) and incubated for 24 hours at 37°C. Clinical isolates presumptively identified as *E. coli* based on a positive reaction on Eosin Methylene Blue Agar (EMBA; Oxoid, Basingstoke, UK) and which showed reduced susceptibility to cefpodoxime (10 μg) and/or cefoxitin (30 μg), used as indicators for ESBL and AmpC production, were selected for this study. In addition, samples from active bacterial environmental surveillance, which is also offered for veterinary hospitals as part of the Diagnostic Service, are also processed by the laboratory, and a cluster of hospital environmental *E. coli* isolates was also included in this study. Detection of resistance to ESC in environmental *E. coli* isolates followed the same protocols as for clinical isolates. The identification of clinical and environmental isolates was performed using API 20E Identification Kits (bioMerieux, France) and also by PCR detection of the *uidA* gene for confirmation of *E. coli.*^24^

### Antimicrobial susceptibility testing

Susceptibility testing was performed by disc diffusion to representatives of beta-lactam and non-beta-lactam antimicrobial classes on ISO-Sensitest agar (Oxoid, Basingstoke, UK) and results were interpreted according to the BSAC (British Society for Antimicrobial Chemotherapy) interpretative criteria.^25^ *E. coli* ATCC 25922 was used as control strain. All isolates were tested for ESBL production by the double disc synergy test (DDST).^26^

### Characterization of ESBL and other resistance genes

Cell lysates obtained from all investigated isolates were screened by PCR and DNA sequencing for the presence of *bla*_CTX-M_, *bla*_SHV_,* bla*_TEM_, *bla*_OXA_, plasmid-mediated *bla*_AmpC_ variants_,_ PMQR genes *qnrA, B, S*_,_ as well as the *cr* variant of *aac(6')-Ib*_,_ as previously described.^27–31^ Specific PCR assays were performed to identify the possible association of *bla*_CTX-M-15_ with IS*Ecp1* or IS*26* insertion elements, which have been shown to be involved in the mobilization and expression of *bla*_CTX-M_ genes.^32,33^

### Resistance transfer and PCR-based replicon typing

To determine the transferability of the ESBL and PMQR genes, conjugation by plate mating was performed with streptomycin-resistant *E. coli* HB101as the recipient and seven selected donors harboring *bla*_CTX-M-15_ (*n* = 6) or *bla*_CTX-M-14_ (*n* = 1). Plasmid replicons involved in the transfer of the resistance genes were analyzed by PCR-based plasmid replicon typing (PBRT) as described by Carattoli *et al.*^34^

### Molecular characterization of isolates

A multiplex PCR described by *Clemont et al*.,^35^ was used to assign the *E. coli* isolates to a phylogenetic group. Genetic relatedness of isolates identified to carry *bla*_CTX-M_ genes was analyzed by macrorestriction pulsed-field gel electrophoresis (PFGE) (www.cdc.gov/pulsenet/pathogens/). Data were analyzed using BioNumerics software version 5.1 (Applied Maths). A tolerance of 1.00% was selected and cluster analysis of PFGE pulsotypes was performed by the unweighted pair group method with average linkages (UPGMA), using the Dice coefficient to analyze similarities and define pulsotypes. PFGE pulsotypes were identified as isolates with ≥90% similarity. Multilocus sequence typing (MLST) was performed as previously described^36^ for at least one isolate from each identified PFGE cluster.

## Results

### Bacterial isolates and antimicrobial susceptibility testing

Four hundred and forty five *E. coli* isolates (*n* = 445) were obtained from companion animal clinical specimens between January 2010 and November 2011, of which 32 (7%) cefpodoxime and/or cefoxitin-resistant nonduplicate isolates (30 canine and two feline) were characterized in this study. The selected isolates were both from normally sterile sites [urine (*n* = 6), liver/bile (*n* = 4), abdominal fluid (*n* = 3), bronchoalveolar lavage (*n* = 1), lymph-node biopsy (*n* = 1)] and also from sites colonized with normal flora and where cultures yielded mixed bacterial growth [colon biopsies (*n* = 3), wounds (*n* = 4), skin/ear swabs (*n* = 5), fecal samples (*n* = 5)]. In addition, a cluster of environmental ESC-resistant *E. coli* isolates (*n* = 6) obtained from the same clinic and during the same time period as two particular ESBL-positive clinical isolates, was also included in the study and characterized by the same methods as the clinical isolates. These two ESBL-positive clinical isolates were from the same dog, which had been admitted with septic peritonitis following duodenal ulceration; one isolate was obtained from abdominal fluid (12L-0659) and one from a surgical site wound swab (12L-0671) following surgery. The environmental ESC-resistant *E. coli* isolates were obtained from the ultrasound table (EBM-111) where the dog was examined and also from various areas of the ward where the dog was hospitalized; these included the kennel area, the drip pump attached to the kennel, the ward door handle, the ward fridge handle, and the ward computer keyboard (EBM-114, EBM-115, EBM-116, EBM-118, and EBM-119).

All isolates characterized in this study showed resistance to ampicillin, amoxicillin–clavulanic acid (CV), cefotaxime, cefpodoxime, ceftazidime, and tetracycline. In addition, 71% of isolates exhibited resistance to cefoxitin, 65% to ciprofloxacin, and 59% to trimethoprim/sulfamethoxazole (Table 1). Interestingly, 29% of isolates showed resistance to amoxicillin-clavulanic acid, but susceptibility to cefoxitin. This may indicate that mechanisms such as those which involve combinations *bla*_CTX-M-15_ and *bla*_OXA-1_ genes (as seen in isolates 10L-4543, 11L-1298, 10L-2646) may give rise to this phenotype. Other mechanisms responsible for amoxicillin-clavulanic acid resistance, but which do not normally confer resistance to the cephamycins includes hyperproduction of TEM-1 or SHV-1 beta-lactamases. Although we did not attempt to determine whether this was the case, a number of the tested isolates carried *bla*_TEM-1b_ only and were fully susceptible to cefoxitin (10L-1747, 10L-2253, 11L-2520, Table 1).

**Table 1.** Phenotypic and Genotypic Characteristics of ESC-Resistant Feline and Canine *Escherichia coli* Clinical and Environmental Isolates (Groupings Based on Common Gene Combinations, PG, and ST, Were Determined)

*Number of isolates (n = 38)*	*Species*	*Isolate ID*	*Source*	*Common antimicrobial resistance profile*	*IS type*	*Beta-lactamase genes*	*PMQR genes*	*PG*	*ST*	*PT*
*n* = 9	Dog	11L-2603	Colon biopsy	AMP, AMC, CPD, CTX, CAZ, FOX, CIP, NA, CN, TE, STX	IS*Ecp1* disrupted by IS*26*	***bla*_CTX-M-15_, _OXA-1, TEM-1, CMY-2_**	*aac-6Ib-cr*	A	410	4
		12L-0659	Wound							4
		12L-0671	Abdominal fluid							4
		EBM (111, 114, 115, 116, 118, 119)	Hospital environment							4 (x6)
*n* = 1	Dog	11L-1050A	Liver biopsy	AMP, AMC, CPD, CTX, CAZ, FOX, CIP, NA, CN, TE, S, STX	IS*Ecp1*	***bla***_**CTX-M-15, OXA-1, TEM-1**_	*aac-6Ib-cr*	D	2348	7
*n* = 2	Dog	10L-3852	Feces	AMP, AMC, CPD, CTX, CAZ, FOX, NA, CIP, CN, TE, S, STX	IS*Ecp1*	***bla***_**CTX-M-15, OXA-1**_	*aac-6Ib-cr*	A		5
		10L-3690	Skin swab							4
*n* = 3	Dog	10L-4543	Skin swab	AMP, AMC, CPD, CTX, CAZ, CIP, NA, CN, TE, S, STX	IS*26* (400bp)	***bla***_**CTX-M-15, OXA-1**_	*aac-6Ib-cr*	B2	131	6
		11L-1298	Bile							6
		10L-2646	Colon biopsy							6
*n* = 1	Dog	11L-0348	Ear swab	AMP, AMC, CPD, CTX, CAZ, FOX, CIP, NA, CN, TE, S, STX	IS*Ecp1*	***bla***_**CTX-M-15, OXA**_	*aac-6Ib-cr*	D	2348	8
*n* = 1	Dog	11L-4755	Feces	AMP, AMC, CPD, CTX, CAZ, FOX, CIP, NA, CN, TE, STX	IS*Ecp1*	***bla***_**CTX-M-15, OXA**_	*aac-6Ib-cr*	A	1284	9
*n* = 1	Dog	10L-1340	Feces	AMP, AMC, CPD, CTX, CAZ, CIP, NA, TE, S, STX	IS*Ecp1*	***bla***_**CTX-M-15**_	–	A	4184	1
*n* = 1	Dog	10L-0827	Abdominal fluid	AMP, CPD, NA, CIP, TE, S, STX	–	***bla***_**CTX-M-27**_		B2	131	7
*n* = 4	Dog	10L-0405/	LN biopsy	AMP, AMC, CPD, CTX, CAZ, FOX, TE, S, STX	–	***bla***_**CTX-M-14, TEM-1**_	–	A	617	3
		10L-0652	Urine							2
		10L-0784(A)	Bile							2
		10L-0784(B)	Bile							
*n* = 1	Dog	11L-2596	Colon biopsy	AMP, AMC, CPD, CTX, FOX, CIP, NA, TE, S	IS*Ecp1*	***bla***_**CTX-M-14, TEM-1**_		A	617	1
*n* = 3	Dog	10L-1747/	Urine	AMP, AMC, CPD, CIP, NA, TE, S	–	***bla***_**TEM-1b**_		A		
		10L-2253/	Urine							
		11L-2520	Skin swab							
*n* = 1	Dog	11L-1050B	BAL	AMP, AMC, CPD, CTX, CAZ, FOX, CIP, NA, CN, TE, S, STX	–	***bla***_**TEM-1, CMY-2**_		D		
*n* = 2	Dog	11L-1345/	Abdominal fluid	AMP, AMC, CPD, CTX, CAZ, FOX, CIP, NA, TE	–	***bla***_**TEM-1, CMY-2**_		A		
		10L-4304	Urine							
*n* = 1	Dog	12L-0098	Urine	AMP, AMC, CPD, CTX, CAZ, FOX, TE, S, STX	–	***bla***_**TEM-1b, CMY-2**_		B2		
*n* = 1	Dog	10L-4532	Feces	AMP, AMC, CPD, CTX, CAZ, FOX, TE, S	–	***bla***_**OXA, CMY-2**_		A		
*n* = 3	Dog	10L-4885/	Urine	AMP, AMC, CPD, CTX, CAZ, FOX, NA, TE	–	***bla***_**CMY-2**_		D		
		11L-0024	Wound infection							
		12L-0372	Feces							
*n* = 1	Dog	10L-3142	Swab	AMP, AMC, CPD, CAZ, FOX, TE	–	***bla***_**CMY-2**_		B2		
*n* = 2	Feline	11L-0677/	Wound infection	AMP, AMC, CPD, FOX, TE, S	–	**–**		B2		
		10L-2129	Wound infection							

The numbers of isolates with a common phenotype and genotype are shown in column 1.

EBM, environmental bacterial monitoring; BAL, bronchoalveolar lavage; LN, lymph node; PMQR, plasmid-mediated quinolone resistance; PG, phylogenetic group; ST, sequence type; PT, pulsotype; AMP, ampicillin; AMC, amoxicillin–clavulanic acid; CFP, cefpodoxime; CTX, cefotaxime; CAZ, ceftazidime, FOX, cefoxitin; CIP, ciprofloxacin; NA, nalidixic acid; CN, gentamicin; TE, tetracycline; S, streptomycin, STX, trimethoprim/sulfamethoxazole.

The eight *E. coli* isolates included in this study for comparison (two clinical and six environmental) were processed in the diagnostic laboratory simultaneously and the identical susceptibility phenotypes identified in this group of isolates triggered closer investigation. In the DDST, they showed no synergy for ceftazidime and cefotaxime with CV combinations, while only a small zone of inhibition (less than 4 mm) appeared for the cefpodoxime/CV combination. All isolates were resistant to cefoxitin and this raised the possibility of the ESBL phenotype being masked by the additional presence of AmpC cephalosporinase, which is not inhibited by CV; additional testing with a cefepime/CV combination revealed the presence of ESBL phenotypes in these eight clinical/environmental *E. coli* isolates.

### Characterization of ESBL and other resistance genes

Among the ESC-resistant clinical *E. coli* isolates, CTX-M type ESBL was the most prevalent, found in 56% (18/32) of isolates, of which 66% (12/18) of isolates harbored *bla*_CTX-M-15_, with *bla*_CTX-M-14_ being found in five isolates (27%) and *bla*_CTX-M-27_ identified in one isolate. With the exception of one isolate, which carried *bla*_CTX-M-15_ alone, the remaining ESC-resistant clinical *E. coli* isolates also carried *bla*_TEM-1_, *bla*_CMY-2_, and/or *bla*_OXA-1_ in various combinations (Table 1). The *aac(6')-Ib*-*cr* gene was the only PMQR gene detected, although at high prevalence (34.3%), and was associated with the carriage of *bla*_CTX-M-15_. The eight clinical and environmental isolates that showed the common ESBL/AmpC phenotype, carried *bla*_CTX-M-15_ and also coharbored *bla*_OXA-1,_ *bla*_TEM-1,_ *bla*_CMY-2,_ and *aac(6')-Ib*-*cr.* Specific PCR assays revealed that IS*Ecp1* or IS*26,* or in some isolates IS*Ecp1* disrupted by IS*26,* was associated with *bla*_CTX-M-15_ (Table 1). In addition, IS*Ecp1*was associated with *bla*_CTX-M-14_ in one of the five isolates and was not found to be associated with *bla*_CTX-M-27,_ findings which support the diversity of the CTX-M genetic arrangements in *E. coli* isolates resulting from various mobilization events.

### Resistance transfer and PBRT

Five transconjugants (four *bla*_CTX-M-15_ and one *bla*_CTX-M-14_), for which the ESBL phenotype and the presence of *bla*_CTX-M-14/15_ was confirmed, were generated on nutrient agar supplemented with cefotaxime (1 mg/L) and streptomycin (50 mg/L). PBRT showed that the transfer of *bla*_CTX-M-15_ was mainly associated with the FIA (*n* = 4), FIB (*n* = 4), IncI1 (*n* = 3), Y (*n* = 2), and B/O (*n* = 1) replicon types, while IncY type replicon was associated with the transfer of *bla*_CTX-M-14._ PCR also showed that *bla*_TEM-1_, *bla*_CMY-2,_ *bla*_OXA__-1,_ as well as *aac(6')-Ib*-*cr*_,_ had cotransferred with the *bla*_CTX-M-14/15_ in all transconjugants, indicating that they are located on conjugative plasmids.

### Molecular typing of isolates

**FIG. 1.**
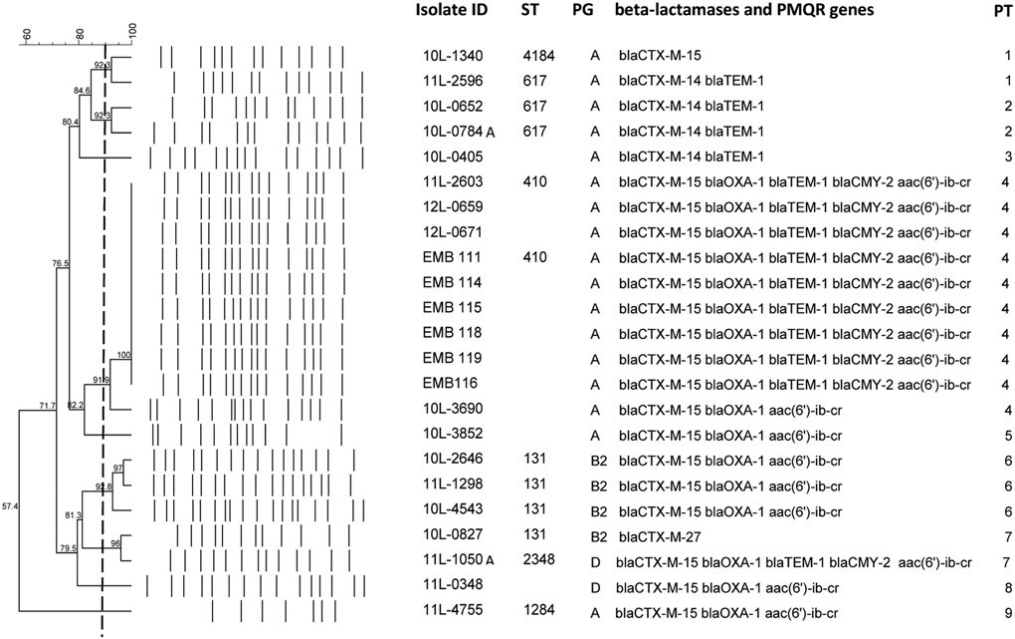
Dendrogram showing cluster analysis of *Xba*I PFGE patterns of CTX-M–producing clinical and environmental (EBM) *Escherichia coli* isolates. The columns to the *right* of the PFGE pattern indicate the ID, ST, PG, and identified beta-lactamases and PMQR genes. PFGE pulsotypes (PT 1–9) were identified as isolates with ≥90% similarity (represented as a *vertical dotted line*). ID, isolate identification; ST, sequence type; PG, phylogenetic group; PFGE, pulsed-field gel electrophoresis; PMQR, plasmid-mediated quinolone resistance.

Phylogenetic typing identified that 63% of *E. coli* isolates belonged to phylogenetic group A and the remaining isolates were typed to the more potentially pathogenic groups, B2 (21%) or group D (15%). PFGE showed clonal diversity of the CTX-M-positive isolates and five pulsotypes (PT 1, 2, 4, 6, and 7) were identified with similarity of isolates greater than 90% (Fig. 1). The main group (PT 4, *n* = 9) included the eight clinical and environmental isolates with the common ESBL/AmpC phenotype and interestingly, another clinical isolate from a colon biopsy obtained from a dog (11L-2603), which was admitted with diarrhea in the same clinic 7 months previously. The second main cluster (PT 6) was formed by three isolates identified to belong to the human pandemic ST131 by MLST. Interestingly, a fourth member of this clone, which carried *bla*_CTX-M-27_, showed only a 73% similarity with the ST131 group. MLST also showed that the clinical and environmental isolates with the common ESBL/AmpC phenotype belonged to ST410, while the next most common ST identified in our *bla*_CTX-M_ isolates was ST617 (Fig. 1).

## Discussion

This study characterized a collection of ESC-resistant *E. coli* and identified a high prevalence (7%) of ESC-resistant *E. coli* in clinical specimens from companion animals in the UK, which is considerably higher than that found in similar studies from pets in France (3.7%) or The Netherlands (2%).^3,10^ We also found a high prevalence (56%) of CTX-M type ESBL-producing *E. coli* from clinical specimens where 66% of clinical ESBL-producing *E. coli* carried *bla*_CTX-M-15,_ which is among the highest reported rates in companion animals. To the best of our knowledge, higher carriage rates of *bla*_CTX-M-15_ in clinical animal isolates have only been reported in the United States where 78% of the ESBL-producing *E. coli* clinical isolates from companion animals were found to carry *bla*_CTX-M-15._^11^ In Europe, 46% and 36% of canine ESBL-producing *E. coli* isolates (from Germany and France, respectively) carried *bla*_CTX-M-15._^10,37^ In The Netherlands, Dierikx *et al.*,^3^ found that of 29 *E. coli* isolates with an ESBL/AmpC phenotype from diseased dogs, cats, and horses, only five isolates (from dogs) (17%) carried *bla*_CTX-M-15._ In addition, a lower prevalence of *bla*_CTX-M-15_ was found in Switzerland, where eight of the 107 *E. coli* isolates obtained from canine urine samples (7.4%) were ESBLs and all carried *bla*_CTX-M-15._^4^ Furthermore, only one *E. coli* isolate carried this gene in a similar study in Italy and no *bla*_CTX-M_ was identified in a study characterizing multidrug-resistant canine urinary *E coli* isolates from Scotland.^38,39^

*E. coli* carrying *bla*_CTX-M-15_ is the most common ESBL type associated with infections in humans in the United Kingdom and Europe.^6^ On this basis, the high prevalence of veterinary clinical isolates carrying *bla*_CTX-M-15_ identified in this study is worrying both in the context of likely interspecies transfer (man to animals), as well as previous studies identifying animals as a potential reservoir of ESBL-producing *E. coli* for human infection.^40,41^ In addition, the clinical and environmental isolates investigated for hospital dissemination had a common ESBL/AmpC phenotype and genotype and MLST showed that they all belonged to ST410. PCR analysis also demonstrated that all isolates had an identical genetic environment of *bla*_CTX-M-15,_ where an IS*26* element was inserted in between *bla*_CTX-M-15_ gene and its promoter found in IS*Ecp1.* PFGE showed 100% homology for the two ESBL/AmpC *E. coli* clinical isolates from the dog with septic peritonitis and the environmental isolates obtained from hospital areas with which this patient came in direct contact (ultrasound table and kennel), or were likely to have spread through staff contact (door handle, the ward fridge handle, and the ward computer keyboard).

This study demonstrated veterinary hospital dissemination of clinical *E. coli* ST410 isolates co-harboring *bla*_CTX-M-15,_ *bla*_TEM-1, OXA-1_ or _CMY-2,_ and *acc(6`)-Ib-cr*, a genotype conferring MDR and often associated with human clinical isolates.^42–44^ Following the confirmation of the ESBL/AmpC phenotype in these isolates, the laboratory contacted the veterinary hospital's infection control team, which took action by cleaning and disinfection of the areas/surfaces identified as sources of these organisms and reinforced hand hygiene policy. The environmental sampling was repeated after reinforcing cleaning and disinfection protocols and no *E. coli* isolates with an ESBL/AmpC phenotype were identified in the subsequent bacterial environmental surveillance specimens. This study demonstrates the role that the microbiology laboratory can play in the early detection and prevention of MDR isolate dissemination in veterinary hospitals. The presence of multiple β-lactamases in Gram-negative bacteria may interfere with the ESBL phenotypic confirmatory tests^45,46^ and it is therefore important that veterinary diagnostic microbiology laboratories are continuously updating their detection methods to recognize ESBL, AmpC, or other emerging resistance phenotypes and to translate the therapeutic or epidemiological significance of these findings to veterinary clinicians. This study also highlights the importance of infection control programs and the benefits of environmental surveillance in the veterinary hospitals for limiting the spread of nosocomial pathogens. Furthermore, the dissemination of the ESBL/AmpC *E. coli* ST410 isolates from veterinary patients (probably from surgical wounds) to the environment, as shown in this study, may indicate a pattern of spread that can occur in the community, especially in the owners home, highlighting the associated human health risk. Therefore, accurate laboratory detection of ESBL/AmpC phenotypes can support the veterinary hospitals in the process of implementing policies for owner's information and infection control advice for limiting the owner's exposure and associated transmission risks.

Recent EUCAST and CLSI guidelines recommend that when using the new interpretative breakpoints, routine ESBL testing is no longer necessary and reporting of susceptibility results to penicillins and cephalosporins for ESBL-producing Enterobacteriaceae should be ‘as found’.^47,48^ However, these new guidelines indicate that ESBL screening may still be useful for epidemiological reasons.^47,48^ Our findings, demonstrating a high prevalence of CTX-M-15 ESBL–producing *E. coli* in clinical specimens from companion animals, as well as the dissemination of *E. coli* ST410 through the hospital environment, support the need for veterinary laboratories to continue ESBL screening and to continuously upgrade their expertise in detection of complex antimicrobial resistance phenotypes, to benefit both human and animal health.
